# First Experimental Application of DNA-Layered Salmonid Alphavirus-Based Replicon Vaccine in Non-Salmonid Fish: Induced Early Semi-Specific Protection against Spring Viraemia of Carp Virus (SVCV) in Common Carp (*Cyprinus carpio*)

**DOI:** 10.3390/ani14182698

**Published:** 2024-09-18

**Authors:** Flóra Abonyi, Edit Eszterbauer, Ferenc Baska, Tímea Hardy, Andor Doszpoly

**Affiliations:** 1 HUN-REN Veterinary Medical Research Institute, H-1143 Budapest, Hungary; 2 Department of Exotic Animal and Wildlife Medicine, University of Veterinary Medicine Budapest, H-1078 Budapest, Hungary

**Keywords:** fish vaccine, replicon vaccine, spring viraemia of carp, gene expression, in vivo vaccination trial, cyprinids

## Abstract

**Simple Summary:**

Common carp is in the top three among the farmed fish species worldwide. Spring viraemia of carp is caused by a virus that is economically significant, causing devastating losses in common carp aquaculture. Hence, the development of new-generation vaccines against this virus is pivotal. Previously, a salmonid alphavirus-based replicon vector system was developed and tested against viruses infecting Atlantic salmon. In our study, we demonstrate the first application of this vector system in a non-salmonid fish species. The replicon system provided significant protection against the spring viraemia virus as early as three weeks post-vaccination.

**Abstract:**

Our study demonstrates the first application of the salmonid alphavirus-based replicon vector system (pSAV) as a DNA vaccine in a non-salmonid fish species, in common carp (*Cyprinus carpio*) against spring viraemia of carp virus (SVCV). SAV replicon encoding the glycoprotein of the SVCV was used as a DNA-layered plasmid, and its efficacy was compared with a previously described conventional DNA vaccine construct (pcDNA3.1 based vector) and with a control group (pcDNA3.1-empty-plasmid) in an SVCV challenge at a water temperature of 14 ± 1 °C. Vaccine prototypes were administered intramuscularly at a dose of 0.1 µg/g of fish (*n* = 25 per group). The DNA-layered SAV replicon resulted in 88% survival, compared to around 50% in all other groups. The DNA-layered pSAV vaccination induced the innate immune genes at the injection site, and increased IgM upregulation was also observed. Our preliminary results show that the SAV-based replicon construct may serve as a potential vaccine candidate for the protection of non-salmonid fish in the future provided that further clinical and field trials confirm its efficiency.

## 1. Introduction

Spring viraemia of carp virus (SVCV) is a negative-strand RNA virus belonging to the genus *Sprivivirus* of the family *Rhabdoviridae*, which infects mainly cyprinids, such as common carp (*Cyprinus carpio*) [1]. It has a considerable economic impact in Central and Eastern Europe, as common carp is one of the most important fish species in this region’s aquaculture. SVCV is often responsible for mass mortalities in fish farming and poses a threat to production [2,3]. The virus infection appears during the spring season (at a water temperature of 10–15 °C) and could cause mortality up to 90%, usually for fingerlings. SVCV is common in Europe [1], but outbreaks have been reported in other regions of the world as well, including the USA and China [4,5], the latter of which supplies more than 70% of the annual carp production worldwide [6]. SVCV is one of the listed aquatic viral diseases of the WOAH (World Organisation for Animal Health) [7]. This cytopathic virus causes acute systemic infection. The common symptoms are skin hemorrhages, exophthalmia, abdominal distension and petechial hemorrhages in the internal organs [1].

One of the most important factors in veterinary health management is reducing mortality caused by infectious diseases with the help of vaccines [8]. There are several DNA vaccines against fish viruses, and in most cases, these vaccines stimulate a broad protective immune response that includes innate and adaptive immunity [9]. CLYNAV was the first DNA vaccine (against salmon pancreas disease) that received marketing authorization from the European Medicine Agency in 2016 [10,11]. In addition, several DNA vaccines against fish rhabdoviruses, such as the viral hemorrhagic septicemia virus (VHSV) and infectious hematopoietic necrosis virus (IHNV), have been developed [12,13,14,15].

A previous study reported that DNA vaccines against SVCV containing the gene of the viral glycoprotein (G) [16] induced moderate to full protection, even in a single low dose, in juvenile carp [2]. While the genome of the SVCV contains five genes expressing structural proteins, the G is an important viral surface protein and has a main role in receptor binding and the induction of viral endocytosis [1,17]. The backbone of these vaccines was the pcDNA3 plasmid, in which the expression of the GOI is under the control of the cytomegalovirus (CMV) promoter. This construct was able to confer up to 100% protection when injected intramuscularly and triggered the local inflammation and B- and T-cell responses [2]. On the contrary, this vector was not able to induce sufficient protection as an oral vaccine; thus, the intramuscular (i.m.) vaccination seems to be the most effective, albeit laborious, method for now [18].

Other promising nucleic acid vaccine constructs against viral diseases are the alphavirus-based replicon vectors. Several vector systems have been designed and tested so far [19]. One of their benefits is these this systems could be applied as RNA replicons or as DNA-layered plasmid constructs. As a self-amplifying DNA vaccine, this type of vaccine contains the cDNA copy of the non-structural genes of the alphaviral genome, which is required for the replication, and the gene of interest (GOI). This system is able to produce an extremely large number of ‘subgenomic’ mRNA copies of the encoded antigen, which is thus under the control of the alphavirus replication machinery [20,21]. The double-stranded RNA intermediates produced in this process have an immunostimulatory effect; in addition, the intracellular antigen expression also induces antiviral responses [21].

Initially, the DNA-layered alphavirus replicons were designed for homeothermic animals, using alphaviruses isolated from mammals (Sindibis virus, Semiliki Forest virus, etc.) [22]. However, the expression rates of these prototypes were significantly reduced when used in fish cells at 15 °C or below [23]. Thus, for aquaculture, the salmonid alphavirus replicon system (SAV) has been developed based on the salmon pancreas disease virus (SPDV) belonging to the genus *Alphavirus,* within the family *Togaviridae*. Studies have shown that the SAV replicon vectors expressed the enhanced green fluorescent protein (EGFP) or luciferase more efficiently than the alphaviruses of mammals, at 15 °C or below, which is the optimal temperature for the replication of salmonid alphaviruses [24,25]. Consequently, the SAV replicons have also been tested in vivo in fish. This vaccine construct has been shown to be effective against infectious salmon anemia (ISA) in Atlantic salmon (*Salmo salar)* delivered i.m., when the replicon expressed the hemagglutinin esterase (HE) [26,27]. However, when a polyprotein-expressing SAV replicon vector system was used to immunize against infectious pancreatic necrosis virus (IPNV), only modest protection was achieved [28].

The SAV replicon has only been tested in vivo in Atlantic salmon [26]; therefore, the aim of our study was to investigate its efficacy in a non-salmonid fish species, the common carp.

## 2. Materials and Methods

### 2.1. Animals and Ethics

The fertilized eggs of common carp were sourced from a cyprinid fish farm at Dinnyés, Hungary and transported to the animal facility of the HUN-REN Veterinary Medical Research Institute (HUN-REN VMRI), Budapest, Hungary, before hatching. Fish were reared under pathogen-free laboratory conditions in an aerated 200 L aquarium at 15–25 °C, up to the age of 3+ years (yrs). Fish were fed with commercial fish pellets (Aller Aqua, then Sera Vipan from yr 1+ supplemented with vitamin C) once a day. The average length of fish was 8 cm during the vaccine trial.

All animal experiments were performed under a license from the Hungarian National Scientific Ethical Committee on Animal Experimentation (PE/EA/00782-6/2023). The minimum number of fish required to obtain statistically reliable results was used. All efforts were made to minimize suffering.

### 2.2. Cells and Virus Strain

EPC (Epithelioma Papulosum Cyprini, ATCC CRL-2872) and KF-1 (Koi fin-1, ECACC 10072801) cells were cultured at 25 °C, and CHSE-214 (Chinook salmon embryo-214, ECACC 91041114) at 21 °C, all in EMEM medium (Lonza, Visp, Switzerland) supplemented with 10% fetal bovine serum (FBS) (Biosera, Nuaillé, France), 1% HEPES buffer (1M) (Biosera, Cholet, France) and 1% penicillin–streptomycin (Lonza, Visp, Switzerland).

The SVCV strain ME/2020 isolated previously in the Molecular Virology laboratory of HUN-REN VMRI was propagated in the EPC cell line at 20 °C [29]. The tissue culture infectious dose (TCID_50_/mL) was determined using the method by Reed and Muench [30].

### 2.3. Vector Constructions and Vaccine Preparation

In the in vitro EGFP expression experiments, the replicon vector pSAV-EGFP, described [27] and kindly provided by Prof. E. Rimstad, was used. For the in vivo fish vaccination experiments, the EGFP gene was replaced with the glycoprotein gene of the SVCV (strain ME/2020) to create a replicon vaccine (pSAV-DNA-SVCV-G). The gene G was amplified from the purified viral RNA using reverse transcription PCR (RT-PCR) (primer sequences forward Fw_SVCV_glycoprotein: CGA CCG GTG CCA CCA TGG CTA TCA TCA GCT ACA T; reverse primer Rev_SVCV_glycoprotein: CGG GCG CGC CTC AAA CTA AAG ACC GCA TTT) and ligated into the replicon vector following digestion with the restriction enzymes AgeI and AscI using the CloneJet PCR Cloning Kit (Thermo Fisher Scientific, Waltham, MA, USA), following the manufacturer’s instructions.

The pcDNA 3.1. vectors were created as follows: the EGFP gene was amplified from the vector pSAV-EGFP (forward primer pcDNA-EGFP_GOI_fo: CGC CGA AGC TTA CCA TGG TGA GCA AGG GCG AGG, reverse primer pcDNA-EGFP_GOI_re: CGC CGG AAT TCT TAC TTG TAC AGC TCG TCC A), whereas the gene G was amplified from the vector pSAV-DNA-SVCV-G (forward primer pcDNA-SVCV-G_GOI_fo: CGC CGA AGC TTA CCA TGG CTA TCA TCA GCT ACA T, reverse primer pcDNA-SVCV-G_GOI_re: CGC CGG AAT TCT CAA ACT AAA GAC CGC ATT). The PCR products were cloned into the pcDNA 3.1. vector with the restriction enzymes HindIII and EcoRI. These constructs were named pcDNA-EGFP and pcDNA-SVCV-G, respectively.

The NucleoBond PC10000 EF plasmid purification kit (Macherey-Nagel, Dueren, Germany) was used for the large-scale plasmid preparation following the manufacturer’s manual, and the concentrations were determined with the Qubit 3 Fluorometer (Thermo Fisher Scientific, Waltham, MA, USA).

### 2.4. In Vitro Gene Expression Assays

CHSE-214, EPC and KF-1 cells were seeded to 24-well plates and incubated at 25 °C. The following day, the cells (70–90% confluence) were transfected with the pcDNA-EGFP and the pSAV-EGFP plasmids using the TurboFect Transfection Reagent (Thermo Fisher Scientific, Waltham, MA, USA). For each well, 1 µg of DNA (pcDNA-EGFP) was added to 100 µL serum-free Opti-MEM medium (Thermo Fisher Scientific, Waltham, MA, USA), mixed thoroughly, then 4 µL of transfection reagent was added and then incubated at room temperature (RT) for 20 min. Subsequently, the mixture was added to the well drop-wise, and the plates were incubated at 25 °C. Six hours later, the medium was changed with fresh EMEM growth medium, and the plates were moved to 15 °C. With the pSAV-EGFP, the same method was used, but 2 µg of DNA and 6 µL of transfection reagent were applied.

The cells were monitored daily with an Axio Observer D1 inverted microscope (Zeiss, Oberkochen, Germany). CHSE-214, EPC and KF-1 cells were incubated for 5, 8 and 14 days, respectively (the number of positive cells peaks at different days after transfection [25]). The growth medium was replaced with FluoroBrite DMEM medium (Thermo Fisher Scientific, Waltham, MA, USA), and the cells were freeze-thawed three times. Subsequently, the fluorescent signal was measured in a Varioscan Lux microplate reader (Thermo Fisher Scientific, Waltham, MA, USA), and the total protein concentration of the samples was determined using the Micro BCA Protein Assay Kit (Thermo Fisher Scientific, Waltham, MA, USA). SkanIt Software 6.1 (Thermo Fisher Scientific, Waltham, MA, USA) was used for the analysis of the microplates (EGFP and BCA).

### 2.5. Preliminary SVCV Challenge

We intended to know the effect vaccine dosage (µg/g of fish) of our virus strain in adult fish with mature immune systems; therefore, the preliminary SVCV infection experiment was performed with a group of 3+ yr carp (n = 10) (average weight 8–12 g), which were infected with 10^6^ TCID_50_/mL SVCV dosage by immersion at 15 °C for 48 h, with an unhandled negative control group, and the mortality was monitored for 30 days post-infection (dpi). The conditions of the SVCV infection were based on the bath challenge previously optimized by Embregts et al. [2,18]. Clinical symptoms were recorded, and liver samples were collected from all fish and stored in an RNAlater solution (ThermoFisher Scientific, Waltham, MA, USA) at −80 °C for subsequent RNA isolation and real-time reverse transcription PCR (RT-qPCR) to confirm virus infection (Table 1).

### 2.6. Expression Analysis of Immune-Related Genes

Gene expression analysis was performed to compare the efficacy of the DNA-layered vaccines (pcDNA-SVCV-G and pSAV-DNA-SVCV-G). Carp were divided into four groups and vaccinated with the two DNA vaccine constructs, whereas the two control groups were injected i.m. with PBS and pcDNA empty plasmid (Table 1) at a fish dosage of 0.1 µg/g, respectively. All groups were kept at 20 °C. Three days post-vaccination (dpv) 3 fish from each of the four groups were euthanized, and muscle samples (at the site of the injection) were collected and stored at −80 °C in RNAlater. Muscle and spleen samples were collected at 6 dpv from the remaining 3 fish from all groups.

Type I interferon (I-IFN) gene expression analysis was performed to follow the interferon activity over 3 weeks after the vaccination with pSAV-G replicon. Carp were divided into 2 groups (n = 6) and vaccinated with pSAV-G i.m. with 0.1 µg/g of fish vaccine dosage, while the control groups were injected with PBS. Fish were kept at 20 °C. At 14 and 21 dpv, 3 fish from both groups were euthanized, and muscle (at the site of the injection), spleen and kidney samples were collected and stored at −80 °C in RNAlater. Blood samples were also collected from all individuals for serum neutralization tests.

### 2.7. Vaccination and SVCV Challenge

Fish were randomly divided into three groups (n = 25 per group in 50 L tanks) and were vaccinated with the following: (i) pcDNA-SVCV-G, (ii) pSAV-DNA-SVCV-G and (iii) pcDNA empty plasmid (negative control), respectively (Table 1). Fish were anaesthetized with 100 mg/L tricaine-methanesulfonate (MS-222, Sigma Aldrich-Merck, Darmstadt, Germany) and vaccinated i.m. with a dosage of 0.1 µg/g of fish vaccine in the epaxial muscle near the dorsal fin. All groups were kept at 20 °C for 17 dpv. Seven days prior to the SVCV challenge, the water temperature was gradually lowered to 14 ± 1 °C at a ratio of 1 °C/day. Thus, the degree-days post-immunization were 452. All groups were infected with SVCV by immersion bath in 6 L tanks with 6 × 10^5^ TCID_50_/mL final virus concentration at 14 °C for 48 h. Subsequently, fish were moved back to the 50 L tanks with aeration and water filtration. The experiment was monitored for 30 days. The moribund and dead fish were documented and removed from the tanks daily. Clinical signs and symptoms were recorded, and the fish were sampled immediately for virus re-isolation. At the end of the experiment, all of the survivors were euthanized with 100 mg/L MS-222 and killed by cranial cut prior to dissection. From all fish, liver samples were collected for RNA extraction and RT-qPCR, and the samples were stored in RNAlater at −80 °C. A small piece of liver from five individuals per experimental group was fixed for histopathological examination.

### 2.8. Virus Re-Isolation

The liver samples were homogenized using a TissueLyser LT disruption instrument (Qiagen, Hilden, Germany) at 50 Hz in 1× TE buffer (pH 8.0) for 5 min. After centrifugation (at 3000× *g* for 5 min), the supernatant of the tissue samples was diluted to 1:100, and 100 µL was inoculated on EPC cells in 25 cm^2^ cell culture flasks. After incubation at 20 °C for 1 h, the inoculum was replaced with EMEM medium with 2% FBS. The cells were checked for the appearance of a cytopathic effect (CPE) daily. The identification of the isolated viruses was confirmed by RT-qPCR.

### 2.9. RNA Isolation

Fish organ samples (liver, spleen and muscles) were homogenized using a TissueLyser LT disruption instrument at 50 Hz for 10 min. Subsequently, the RNA was isolated from the homogenates with an RNeasy Mini Kit (Qiagen, Hilden, Germany), according to the instruction manual. The extracted RNA samples were stored at −80 °C.

### 2.10. Real-Time Reverse Transcription PCR (RT-qPCR)

Following the RNA isolation, RT-qPCR was performed on the samples from the preliminary SVCV challenge and on the samples of the surviving fish from all vaccine trial groups to examine the presence of viral RNA. The RT-qPCR (SYBRgreen) assay was performed using specific real-time PCR primers for the SVCV-*n*-gene, as described previously (SVCV-n forward primer: TGA GGT GAG TGC TGA GGA TG; SVCV-n reverse primer: CCA TCA GCA AAG TCC CGG TAT) [2].

In the gene expression analysis, the immune genes tnfα, ifnφ1, ifnγ2a/2b, zap70, IgM were examined with specific primers [2], and β-actin was used for normalization. Relative expression of the SVCV-G gene was analyzed with specific primers [31] to confirm the expression of the G-protein from the vectors in the injection site and to compare their expression levels at 3 days post-vaccination.

The relative expression of the I-IFN was analyzed with specific primers [32] on the samples collected at 6, 14 and 21 dpv from the PBS- and pSAV-DNA-SVCV-G-injected groups.

All RT-qPCRs were performed with SensiFAST SYBR Hi-ROX One-Step Kit (Bioline, London, UK), and the mixture contained 5 µL of One-Step Mix (2×), 0.9 µL of DEPC-H_2_O, 0.1 µL of reverse transcriptase, 0.2 µL of RNase Inhibitor (10 U/µL), 0.4 µL of forward and reverse primers (10 pmol/µL), and 3 µL of total RNA template (ca. 4–7 µg/µL). The program consisted of an initial reverse transcription at 45 °C for 10 min, followed by 95 °C for 2 min and 40 cycles of 95 °C for 5 s and 65 °C for 30 s, carried out in a CFX96 Real-Time PCR System instrument (Bio-Rad, Hercules, CA, USA). All RT-qPCRs were performed in duplicates. The results were analyzed with the CFX Maestro 1.1 software (Bio-Rad).

### 2.11. Sequence Analysis

Sanger DNA sequencing was performed on all of the vaccine constructions using a BigDye Terminator v3.1 Cycle Sequencing Kit (Thermo Fisher Scientific, Waltham, MA, USA), and the detection was carried out on an ABI Prism 3100 Genetic Analyzer (Thermo Fisher Scientific, Waltham, MA, USA) by a commercial service provider. The DNA sequences were analyzed with the BioEdit [33] and Geneious Prime (Biomatters Ltd., Auckland, New Zealand) software packages, and sequence identity was determined using a BLASTn algorithm on the NCBI website.

### 2.12. Histological Examination

At the end of the experiment, liver samples were collected from 5 fish from all groups for histology. The pieces of organs were placed in 8% neutral-buffered formalin (NBF) for 24 h. After fixation, the liver samples were embedded in paraffin wax, cut into 4−5 µm sections and stained with hematoxylin and eosin.

### 2.13. Serum Neutralization Test

The blood samples were kept at RT for one hour, then allowed to clot at 4 °C for 24 h. The sera were separated and collected by centrifugation (1000× *g*, 4 °C, 10 min). Subsequently, two-fold dilutions of the sera were prepared and mixed with SVCV (final titer: 100 TCID_50_) and incubated at 20 °C for one hour. Then, the mixtures were dispersed into 96-well plates with EPC cells (80–90% confluence) and incubated at 20 °C for one hour. After the mixtures were replaced with MEM medium with 2% FBS, the cells were incubated for one week at 20 °C and were evaluated under a light microscope.

### 2.14. Statistical Analysis

Statistical analysis was performed for the EGFP expression, SVCV challenge and gene expression analysis. All the data were analyzed using R Commander (version i386 4.1.1.) (Vienna, Austria) software. In the EGFP expression experiment, the expression data of the two vectors were compared using Student’s *t*-test. In the SVCV challenge, survival data of the three groups were statistically compared using Fisher’s exact test, and graphs were generated with Microsoft Excel. The Relative Percentage Survival (RPS) was determined as a level of protection by the vaccine. RPS was calculated following the formula: RPS = [1 − (% mortality of treated group)/(% mortality of control group)] × 100%.

For gene expression analysis, ΔΔCt values were calculated. Principally, the gene expression data of the two control groups (PBS and pcDNA-empty-plasmid) were compared using Student’s t-tests. Significant differences (*p* < 0.05) between the vaccinated and the control groups at the specified time point were determined by Welch’s ANOVA, followed by Dunnett’s test. The expression levels of the G-protein in the two vaccinated groups were compared using Student’s t-test. In case of the IFN-I analysis, PBS and pSAV-DNA-SVCV-G groups were compared using Student’s t-tests at the specified time point. Among the samples collected at different time points (6, 14 and 21 dpv), significant differences (*p* < 0.05) were determined by Welch’s ANOVA, followed by Tukey’s test. Graphs were generated by Origin Pro software (2023b).

## 3. Results

### 3.1. In Vitro Gene Expression Assay

**Figure 1:**
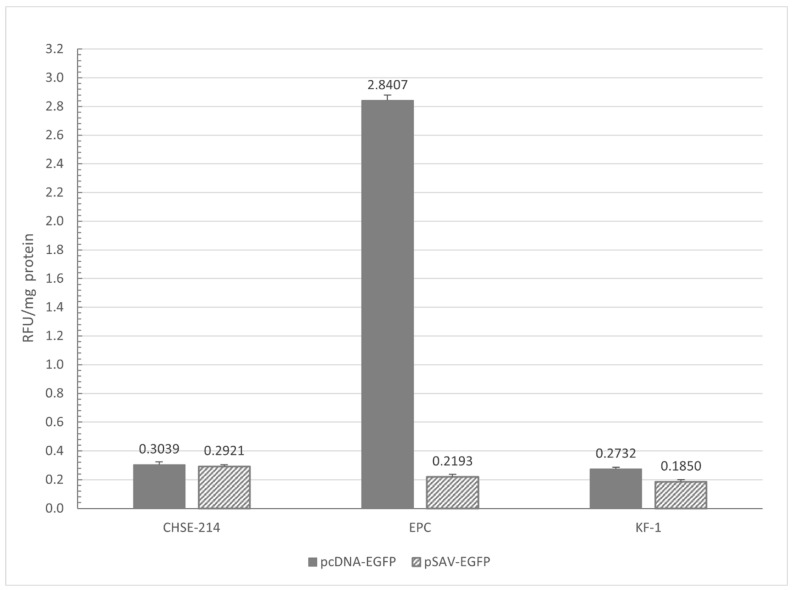
EGFP expression in the CHSE-214, EPC and KF-1 cell lines: EGFP expression was quantified in the CHSE-214, EPC and KF-1 cell lines and normalized to total mg of protein. Values are expressed as the mean +/− SEM. RFU: relative fluorescent unit.

No CPE was observed in the cells transfected with pSAV-EGFP, as compared to those transfected with pcDNA-EGFP. EGFP was detected in pcDNA-EGFP-transfected cells (in all cell lines) after 24 h, while it was detected only after 2–3 days in the pSAV-EGFP-transfected cells. The number of the fluorescent cells peaked around 5 days after post-transfection (dpt) in the CHSE-214 cells and around 8 and 14 dpt in the EPC and KF-1 cells, respectively. The efficacy of the transfection with the pcDNA vector in EPC cells was sufficient, as a fluorescence signal was detected in approximately 80% of the cells. However, in the other two cell lines, the transfection efficacy was only around 10–20%. Using the large (approximately 12 kb) pSAV plasmid, the transfection efficacy was similarly low (10–20%) in all the three cell lines. The difference between the level of the gene expressions of the pcDNA-EGFP and the pSAV-EGFP vectors was not significant in the case of the salmon (CHSE-214) and carp (KF-1) cell lines (Figure 1). In the EPC cells, the EGFP signal produced by the pcDNA vector was almost ten times higher than that of the pSAV vector (Figure 1). The difference was highly significant in this case (*p =* 0.00013).

### 3.2. Preliminary SVCV Challenge

**Figure 2:**
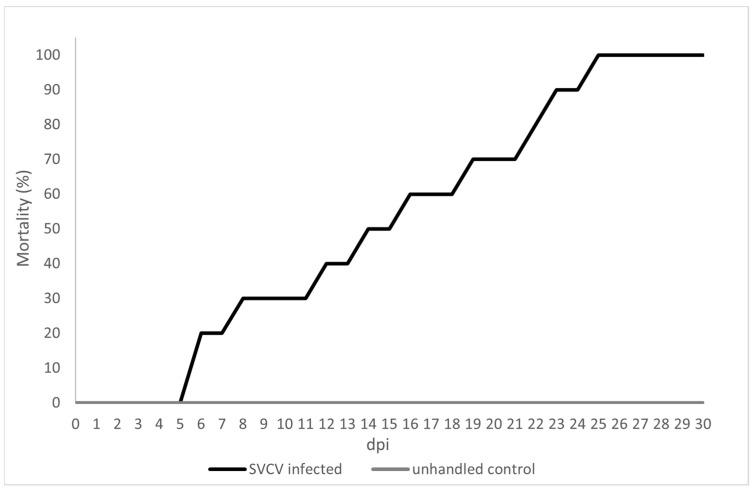
Preliminary SVCV immersion bath challenge: 3+ yr carp (*n* = 10/group) were challenged with SVCV by immersion bath (10^6^ TCID_50_/mL final virus concentration) at 15 °C for 48 h, with a negative control group. Mortality was recorded from 0 to 30 dpi; 100% mortality was observed in the infected group 24 dpi.

The preliminary SVCV infection experiment resulted in 100% mortality in the 3+ yr carp infected with 10^6^ TCID_50_/mL by immersion, and no mortality was observed in the control group (Figure 2). The necroscopy showed the clinical signs of the SVCV infection: skin hemorrhages, exophthalmia, abdominal distension and petechial hemorrhages in the internal organs. The SVCV-specific RT-qPCR resulted in 100% PCR positivity of the samples.

### 3.3. Immune-Related Gene Expression Analysis after Vaccination

The expression of the G-protein in the injection site was confirmed and no significant difference was found in the expression level between the vaccinated groups (*p* = 0.9245). Mean ΔΔCt values: pcDNA-SVCV-G: −12.9144 and pSAV-DNA-SVCV-G: −13.1623.

**Figure 3:**
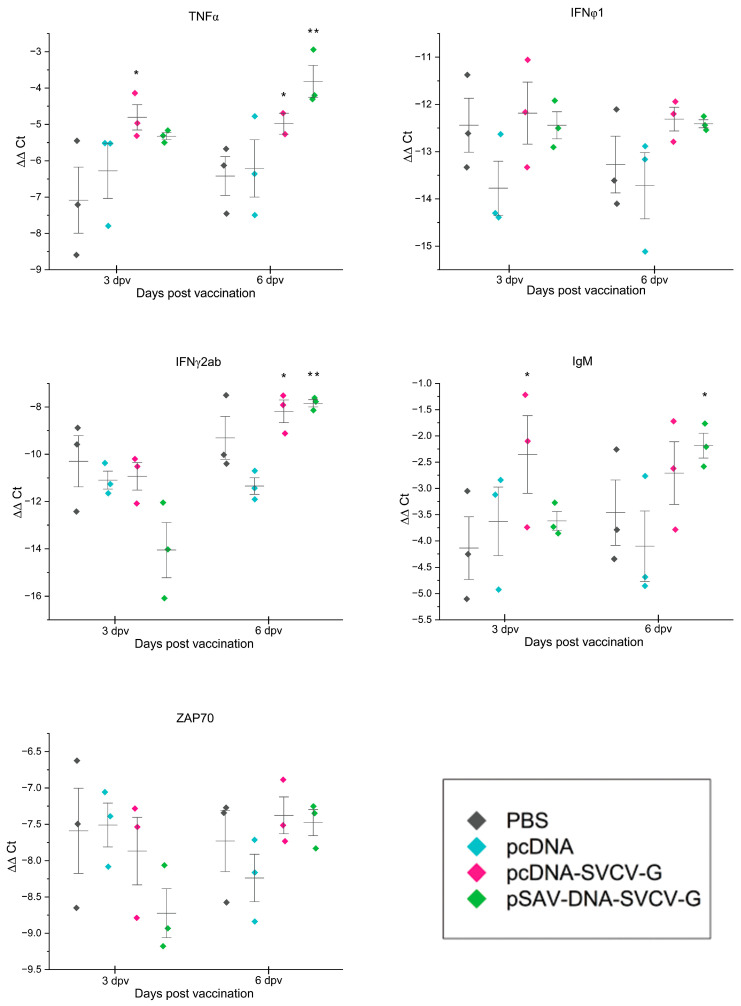
The relative expression levels of immune-related genes: the relative expression levels of genes tnfα, ifnφ1, ifnγ2a/2b, zap70, IgM in muscle (3 dpv) and in both muscle and spleen tissues (6 dpv) of the fish after immunization with pcDNA-SVCV-G and pSAV-DNA-SVCV-G, as well as control groups, which were injected with PBS and pcDNA empty plasmid. Fish muscles were sampled at the site of injection at 3 and 6 days post-i.m. vaccination. Analysis was performed with RT-qPCR and normalized to the housekeeping gene β-actin, and ΔΔCt values were calculated. Statistically significant differences: ** *p* < 0.05, * *p* < 0.1, between the DNA-vaccinated and the control groups determined by one-way ANOVA, followed by Dunnett’s test.

For the pcDNA-SVCV-G vaccine group, an upregulation trend was observed in the relative expression of tnfα, ifnφ1, ifnγ2a/2b, and IgM, usually after only 3 days, and the mean values were nearly the same until 6 dpv. However, none of these expression levels was statistically significant compared to the control groups (Figure 3). In the pSAV-DNA-SVCV group, the relative expression levels of tnfα, ifnγ2a/2b and IgM were higher at 6 dpv, and the difference was significant for tnfα (*p* = 0.0055) and ifnγ2a/2b (*p* = 0.0344) compared to the control groups.

### 3.4. Expression Analysis of Type I Interferon Gene Activity

**Figure 4:**
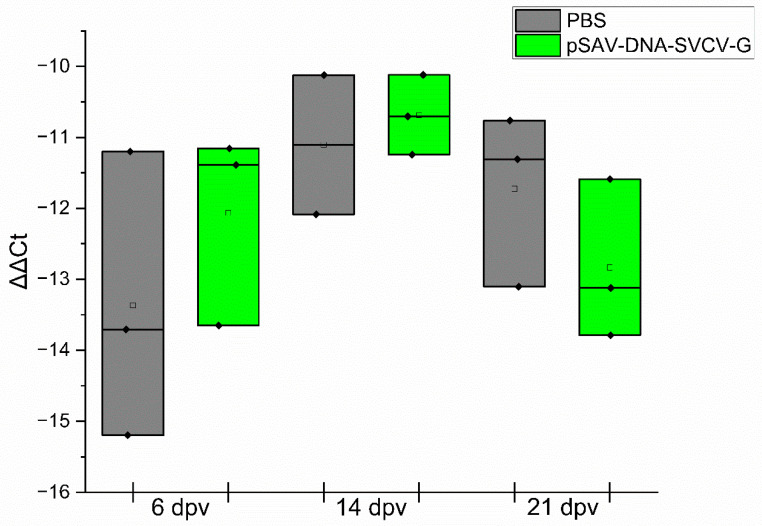
The relative expression levels of type I IFN gene: The relative expression levels of type I IFN gene in muscle, kidney and spleen tissues (6, 14, 21 dpv) of the fish after immunization with pSAV-DNA-SVCV-G, as well as control groups, which were injected with PBS. Fish muscles were sampled at the site of injection at 6, 14 and 21 days post-i.m. vaccination. Analysis was performed with RT-qPCR and normalized to the housekeeping gene β-actin, and ΔΔCt values were calculated. The PBS and pSAV-DNA-SVCV-G groups were compared using Student’s t-tests at the specified time point. The data from the samples collected at different time points were analyzed by one-way ANOVA, followed by Tukey’s test. The difference was considered significant at a *p*-value less than 0.05.

For the pSAV-SVCV-G vaccine group, an upregulation trend in the relative expression of I-IFN was observed 2 weeks after vaccination, but it was not statistically significant compared to the values at the other sampling times (Figure 4). No significant difference was found between the groups at the specified time points.

### 3.5. Serum Neutralization Test

There was no observable neutralizing effect in the control group. In the vaccinated group, neutralization was detected; however, the neutralizing antibody titers were rather low (1:2 dilution).

### 3.6. Vaccination and SVCV Challenge

**Figure 5:**
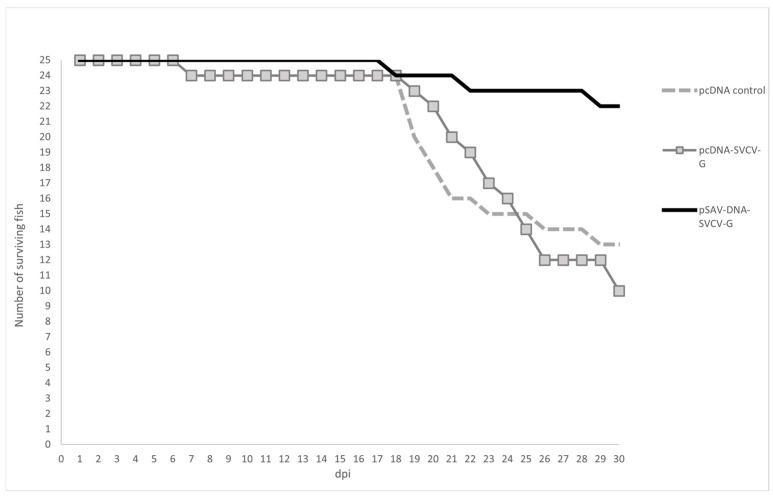
Vaccination and SVCV challenge: three-year-old common carp (n = 25/group) were vaccinated with the pcDNA empty plasmid control, pcDNA-SVCV-G or pSAV-DNA-SVCV-G at 20 °C (the dosage of 0.1 µg/g fish). Three weeks post-vaccination, the groups were challenged with SVCV by immersion bath with 6 × 10^5^ TCID_50_/mL final virus concentration at 15 °C for 48 h. Survival rate was recorded from 0 to 30 dpi.

Using the data from the preliminary infection, which demonstrate the susceptibility of the 3+ yr carp to the SVCV strain ME/2020, DNA and RNA vaccination and the follow-up SVCV challenge resulted in differences among groups (Figure 5). Similar mortality rates were observed in the pcDNA empty plasmid control (48%) and the pcDNA-SVCV-G (60%) groups; the difference between these groups was not statistically significant. However, a high level of protection was found in the group immunized with the pSAV-DNA-SVCV-G replicon vaccine; the mortality (12%) was significantly lower (*p* = 0.0011) compared to the other groups. Thus, the pSAV-DNA vaccine resulted in the best survival rate, as the RPS was 75%, at 452 degree-days post-immunization.

The common clinical signs caused by the virus were noticed mainly in the dead fish. Gross lesions included petechial hemorrhages in the skin, gills and eyes, and internally, hemorrhages, ascites and catarrhal enteritis were usually seen. Exophthalmia was also notable in the surviving fish. Virus re-isolation from the dead fish from all groups was successful in all cases, a definite CPE was observed on EPC monolayer inoculated with the homogenized liver samples at 2 dpi, and CPE was complete 2 days later. RT-qPCR and the DNA sequence analysis confirmed that the SVCV strain used for fish exposure was isolated. SVCV-specific RT-qPCR performed in all of the surviving fish from all groups confirmed the presence of the virus.

### 3.7. Histopathology

**Figure 6:**
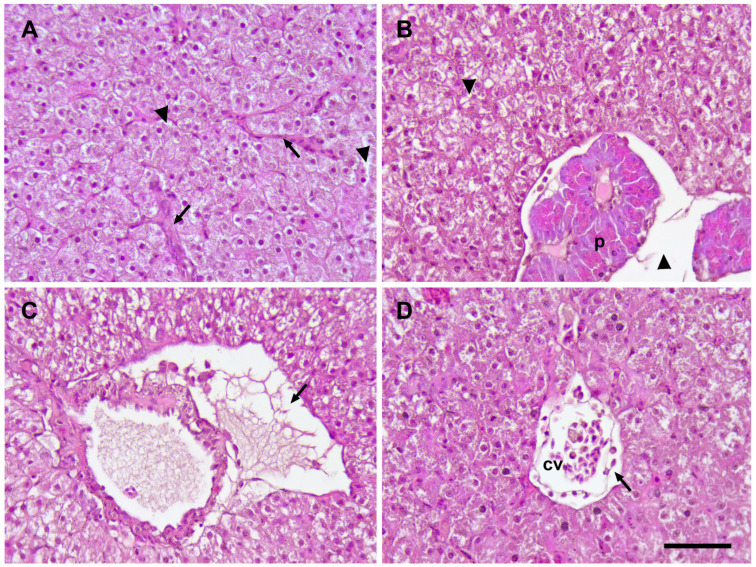
Histopathological changes in the liver of the control and vaccinated groups of common carp exposed to SVCV: (**A**) hepatitis (congested vessels—arrow) and edema (arrow head) in the liver of control fish; (**B**) inflammatory edema (arrow head) in liver parenchyma, while pancreas (p) remained intact (KT1); (**C**) perivascular infiltration with blood serum around the vein (arrow) (SDT4); (**D**) vacuolated and damaged endothelial cells (arrow) of the central vein (cv) and perivascular infiltration (SRT5). H&E staining. Scale bar: 50 µm.

Fatty infiltration of the liver was detected in all fish groups (probably due to the fish pellet diet). Notable histopathological differences were not observed among the experimental groups (Figure 6). Presumably, the virus-induced changes were more pronounced in the control group, where hepatomegaly and hepatitis were observed (Figure 6A,B). Lymphohistiocytic, perivascular infiltration was detected in both control and vaccinated groups. The endothelial cells were vacuolated and partially destroyed, and the blood cells passed beyond the capillaries and accumulated in the surrounding tissue (Figure 6C,D). The liver samples from the vaccinated groups were slightly more homogeneous, with less edema.

## 4. Discussion

In the present study, we provide the first data about the application of the SAV replicon vaccine technology in non-salmonid fish, namely in the common carp, which is in the top three among the farmed fish species worldwide [3]. SAV replicon expressing the G-protein of SVCV was designed, and its efficacy was compared with the previously described conventional DNA vaccine construct (pcDNA3-SVCV-G). The SAV replicon was used as a DNA-layered plasmid. Gene expression analysis and serum neutralization were performed to measure the immune responses after the vaccination of the fish, while the protective effects of the vaccines were tested in an SVCV challenge.

First and foremost, we demonstrated in an in vitro study that the pSAV replicon is able to express the EGFP in a cell line originated from carp. Former studies have already demonstrated its suitability for use in bluegill (*Lepomis macrochirus*) and fathead minnow (*Pimephales promelas*) cells [23,25]. Although in our study, the efficacy of the transfection was far from optimal, the transfection of non-salmonid cells by the pSAV vector resulted in a similar expression level of the reporter gene to that observed in the salmonid cells. This gene expression was much slower than that achieved by the pcDNA vector, a phenomenon that expected based on previous studies [26,34].

Previously, several studies investigated the susceptibility of carp to different strains of SVCVs, the optimization of the virus challenge models, and the efficacy of DNA vaccine constructions [2,18,35]. During the standardization of the SVCV bath-challenged model, more than 90% mortality was observed by Embregts et al. at 15 °C in juvenile carp, applying longer exposure time (48 h). In addition, age-related mortality was detected, and no mortality was recorded in older, more than 9-month-old carp (20 g) [2]. We used more carp that were more than three years old but relatively small (8–12 g) for infection trials, and the preliminary experiment (for 48 h at 15 °C using a dose of 10^6^ TCID_50_/mL of SVCV dose) resulted in 100% mortality, showing that age-related susceptibility is not as obvious as previously thought. Comparing these data could be difficult due to the large contrast in fish age. The similar mortality observed in these studies might be the consequence of the different virus strains and the susceptibility of the different common carp stocks, as the temperature and the exposure time were the same. It is likely that viral mortality is also related to the size or the weight of the fish. 

Regarding the protection of the pSAV replicon and pcDNA-based vector construct, our vaccination protocol followed the previously investigated lower dose of pcDNA plasmid (0.1 µg/g of fish), which was already tested in rainbow trout (*Oncorhynchus mykiss)* against IHNV [36] and in common carp against SVCV [2]. In our experiment, the pcDNA-SVCV-G vaccine did not show protection; thus, our result differed from that of the previous study [2], although approximately the same mortality rate was observed as in the control group. The main difference in our vaccination protocol was the shorter time period (i.e., 3 weeks) between the vaccination and the infection, instead of the previously described 2.5 months [2]. A good representation of the difference between the two protocols is the value of the ‘degree-days’. In our experiment, 452 degree-days was considerably less than the previously recommended value, which was around 1500–1800. During these periods, fish were kept at 20 °C. The higher temperature is known to be an important factor for the T-helper cell and antibody formation, and the delayed development of the neutralizing antibodies could already be observed at 10 or 15 °C compared to 20 °C [37]. In addition, it is known that the CMV promoter, which controls the GOI in pcDNA, is more effective at 20–25 °C [25]. Our RT-qPCR results showed that the expression rate of the G-protein gene in the injection site was the same for both the pcDNA and pSAV vaccines. This also supports our hypothesis, that the shorter exposure time at 20 °C could cause a decrease in protection in case of pcDNA.

In contrast to the conventional DNA vaccine, fish vaccinated with the pSAV-SVCV-G replicon had a significantly higher survival rate of 88% in the SVCV challenge (RPS = 75%). The pSAV replicon systems are able to express proteins more effectively at lower temperatures, as it was described both in vitro [24,25] and in vivo in Atlantic salmon [26,27]. The pSAV/HE replicon, which achieved the 84% RPS_endpoint_ in the ISAV challenge, was also tested previously at 12 °C [27]. The lower temperature is crucial for virus replication, and similar to ISAV, lower temperature (15 °C) is optimal for SVCV. In the same way, the salmonid alphavirus replicon systems, due to the characteristics of the alphavirus, were also adapted to lower temperatures. The optimal temperature range of the SVCV and the vaccine overlapped, which could be a reason why the SAV replicons were more successful, and our results showed that the SAV replicon expressing the G-protein is able to protect common carp against SVCV as early as 3 weeks post-vaccination.

Previous studies reported that long-term histopathological changes were not observed in fish tissues after DNA vaccination [38,39]. However, the histological changes in the tissues of vaccinated fish have not been studied much, and immunohistochemistry for the SAV replicon has been performed only on fish muscle previously [26,27]. The reduction in clinical signs in the internal organs was only observed with an inactivated whole virus vaccine and a DNA vaccine against pancreas disease [40,41,42]. In these studies, differences were found in the heart and pancreas tissue of Atlantic salmon, whereas in our study, pathological changes were also observed in the liver of the vaccinated groups, although to a lesser extent compared to the control group.

Alphaviral replicons are known to induce strong innate immune responses, activate the IFN responses and the signaling pathways by the transfected apoptotic cells and APCs. In addition, the generated double-stranded RNA intermediates can enhance the immunogenicity and efficacy in mammals [22]. Similar responses were observed in Atlantic salmon immunized with SAV-based vaccines [26]. The pSAV/HE vaccine could induce interferon and interferon-regulated gene expression, the IFN-α, RIG-I, and the MX, Viperin and CCL19, locally, in the muscle [26,27]. The upregulation of the innate immune genes is often a consequence observed in fish vaccinated with pcDNA, and in these cases, cytokines are also upregulated effectively [2]. In our case, the pcDNA-SVCV-G resulted in a noticeable (although not significant) overexpression of TNFα and IFNφ, usually occurring earlier and already observable at 3 dpv, whereas the pSAV-SVCV-G showed a significant upregulation of these genes later, at 6 dpv. Based on the previous and our results, the stronger responses of the pro-inflammatory genes may be the consequence of the replicon vector itself. However, stress, any other plasmid backbone and the damages of the injection could also result in inflammation. The upregulation of the adaptive immune genes, such as IgM and the IFNγ, which was found to modulate T-cell proliferation and enhance antigen-specific responses in carp, was observed previously in the case of the pcDNA-SVCV-G vaccine [2]. We also observed a slight upward trend in IgM expression; nevertheless, pSAV-SVCV-G resulted in higher IgM mean expression values and significant IFNγ induction. These could be a sign of a specific immune response, although we did not observe an increase in ZAP70 expression. Although neutralizing activity was found in the serum samples of fish from the vaccinated group, the titers were very low; hence, drawing conclusions from these results would be elusive. In a previous study, when pSAV replicon-based vaccine was used successfully against ISAV, similarly low neutralization titers were reported [26]. It is known that the induction of I-IFN and other innate mechanisms after DNA vaccination may enhance non-specific protection at the presumed time of EARV (early antiviral response) [43,44]. In this study, the I-IFN gene expression analysis showed that the I-IFN level peaked slightly after 2 weeks of vaccination with pSAV-DNA-SVCV-G, although a statistically significant difference was not detected, compared to the PBS group, indicating that it might be triggered by the damage of the injection. Since the challenge was performed at 3 weeks post-vaccination, we could not exclude that the observed protection might be due to innate mechanisms. The pSAV replicon (encoding EGFP or viral protein) could trigger the genes of EARV in the muscle of Atlantic salmon at 8 dpv [27]; however, we could not observe significant I-IFN upregulation at 21 dpv, when the challenge was performed. Indeed, the innate immunity triggered by the vaccine had an apparent role in the protective effect of the pSAV-DNA-SVCV-G, but the results in the I-IFN expression at 21 dpv and the serum-neutralizing effect also suggest that the adaptive immunity was activated in the challenge. Further examination using next-generation sequencing techniques (i.e., high-throughput gene expression assay) could help to understand the background of the immune response mechanisms to the pSAV-based replicons.

## 5. Conclusions

Our first trial and preliminary results show that the SAV-based replicon may serve as a potential vaccine candidate for non-salmonid fish in freshwater aquaculture in the future if further clinical and field trials confirm its efficiency.

## Figures and Tables

**Table 1:** Experimental design of the preliminary SVCV challenge, SVCV vaccination trial and gene expression analysis: dosage, application, group number, sampling and examinations.

Groups	Vaccine Dosage (µg/g of Fish)	Vaccine Application	Group Number (N)	Samples, Time Points	Examination
**Preliminary SVCV challenge**					
Infected	-	-	10	dead fish: liver; on the day of death	RT-qPCR
Unhandled control	-	-	10	-	-
**SVCV vaccine trial**					
pcDNA-SVCV-G pSAV-DNA-SVCV-G pcDNA empty plasmid	0.1	i.m.	25	dead fish: liver; on the day of death survivors: liver; at the end of the experiment *	virus re-isolation RT-qPCR, histology
**Gene expression analysis**					
pcDNA-SVCVG pSAV-DNA-SVCV-G pcDNA empty plasmid	0.1	i.m.	6	3 fish (3 dpv): muscle 3 fish (6 dpv): muscle and spleen *	RT-qPCR
PBS	20 **				

* For all groups ** µL; i.m.: intramuscular.

## Data Availability

The original contributions presented in this study are included in the article; further inquiries can be directed to the corresponding author/s.
